# IMMEDIATE AND LATE RESULTS OF ILEOSTOMY CLOSURE IN PATIENTS WITH FAMILIAL ADENOMATOUS POLYPOSIS UNDERGOING RESTORATIVE PROCTOCOLECTOMY BY OPEN OR LAPAROSCOPIC APPROACHES

**DOI:** 10.1590/S0004-2803.24612025-017

**Published:** 2025-07-21

**Authors:** Fábio Guilherme CAMPOS, Carlos Augusto Real MARTINEZ, Carlos Frederico Sparapan MARQUES, Ulysses RIBEIRO, Paulo HERMAN

**Affiliations:** 1 Departamento de Gastroenterologia, Staff do Serviço de Cirurgia do Cólon, Reto e Ânus da Disciplina de Coloproctologia, Hospital das Clínicas (HCFMUSP), São Paulo, SP, Brasil.; 2 Universidade de São Francisco, Bragança Paulista, SP, Brasil.; 3 Departamento de Gastroenterologia, Diretor do Serviço de Cirurgia do Cólon, Reto e Ânus da Disciplina de Coloproctologia, Hospital das Clínicas (HCFMUSP), São Paulo, SP, Brasil.; 4 Departamento de Gastroenterologia, Professor Titular das Disciplinas de Cirurgia do Aparelho Digestivo e Coloproctologia, Hospital das Clínicas (HCFMUSP), São Paulo, SP, Brasil.

**Keywords:** Adenomatous polyposis coli, restorative proctocolectomy, laparoscopy, Polipose adenomatosa familiar, proctocolectomia restauradora, laparoscopia

## Abstract

**Background:**

Restorative proctocolectomy (RPC) is a common surgical indication to manage familial adenomatous polyposis (FAP) patients.

**Objective:**

We compared outcomes after ileostomy closure in patients undergoing laparoscopic (LAP) or conventional (OPEN) RPC at one single institution.

**Methods:**

Charts from FAP patients (1997-2013) were reviewed. Demographic data (age, sex, previous surgery) and surgical outcomes (original surgical approach, early and late morbidity, complications and reoperations after ileostomy closure) were compared.

**Results:**

A total of 84 patients (53 women and 31 men) submitted to ileostomy closure at a mean age of 30.6 years (11-64) were analyzed. Twenty-one (25%) and 63 patients (75%) formed the OPEN and LAP groups, respectively. Demographic features were similar. After pouch construction, 27 early (32.1%) and 8 late (9.5%) complications occurred, with no mortality. Although overall morbidity rates were similar between both approaches, late complications rate were less common in LAP patients (7.9% x 14.2%). After ileostomy closure, complications were registered in 6 (7.1%) patients, and patients previously operated with the LAP approach also presented less complications (4.7% x 14.2%) and reoperations (3.1% x 9.5%). Additionally, the need for surgical management of complications was greater in the OPEN (9.5%) than the LAP group (3.1%). Besides these numbers, analysis didn’t reveal statistical differences among both groups.

**Conclusion:**

In the conditions of the present manuscript, the abdominal approach used for restorative proctocolectomy doesn’t seem to decisively influence outcomes after loop ileostomy reversal. In the future, analysis of a greater number of patients may probably demonstrate an expected greater complication and reoperation rates in those previously treated through OPEN procedures.

## HIGHLIGHTS

• Ileostomy closure is an important part of the surgical treatment of FAP patients undergoing restorative proctocolectomy by any approach.

• Complication rates after loop ileostomy reversal occurred in 7% of a group of 84 FAP patients.

• Among those operated with the laparoscopic approach, complications (4.7% x 14.2%) an reoperations (3.15% X .5%) were less common when compared to the group treated with conventional approach.

• In the future, annalysis of a greater number of patients may probably reveal an statistical difference between these numbers, thus clearly demonstrating this great advantage of minimally invasive procedures in this group of patients.

## RESUMO

**Objetivo:**

Este estudo comparou os resultados após fechamento de ileostomia em pacientes com polipose adenomatosa familiar (PAF) submetidos a proctocolectomia restauradora (PCR) laparoscópica (LAP) ou convencional (OPEN) em uma única instituição.

**Métodos:**

Pacientes operados entre 1997-2013 foram revisados. Dados demográficos (idade, sexo, cirurgia prévia) e resultados cirúrgicos (acesso cirúrgico original, complicações precoces e tardias, complicações e reoperações após fechamento de ileostomia) foram comparados entre os grupos LAP e OPEN.

**Resultados:**

Foram identificados 84 pacientes (53 mulheres e 31 homens) com idade média de 30,6 anos (11-64). Vinte e um pacientes (25%) e 63 (75%) formaram os grupos OPEN e LAP, respectivamente. Ambos tinham características demográficas similares. Após PCR, houve 27 complicações precoces (31,1%) e 8 tardias (9,5%), sem mortalidade. Embora o índice geral de morbidade seja similar entre LAP e OPEN, as complicações tardias foram menores após LAP (7,9% x 14,2%). Após fechamento de ileostomia, registraram-se complicações em 6 (7,1%) doentes, em que os operados via LAP também desenvolveram menos complicações (4,7% x 14,2%) reoperações (3,1% x 9,5%). A necessidade de manejo cirúrgico dessas complicações foi maior no grupo OPEN (9,5% x 3,1%). Apesar dessas grandes diferenças numéricas, a análise estatística não revelou diferenças significativas devido aos reduzidos números em cada grupo.

**Conclusão:**

Nas condições do presente estudo, o acesso abdominal utilizado para a proctoclectomia restauradora não parece influenciar decisivamente a evolução após fechamento de ileostomia em alça. No futuro, a análise de um maior número de paciente provavelmente poderá demonstrar um esperado maior número de complicações e reoperações entre aqueles previamente tratados por procedimentos abertos.

## INTRODUCTION

After the first report back in 1966, loop ileostomies turned to be increasingly performed^1^. This procedure has been associated with important advantages such as smaller volume under clothes, lack of odor, liquid discharge, reduced rates of parastomal hernia and prolapse. Moreover, there is an expected decrease in complications when compared to colostomies^2^.

Especially after colorectal, coloanal and ileoanal anastomosis for the treatment of cancer, inflammatory bowel disease and familial adenomatous polyposis (FAP), the creation of a diverting protective ileostomy has been advocated to prevent anastomotic leak and mitigate consequences in patients with increased risks and need for interventional or surgical revisions. Moreover, this procedure simultaneously adds little morbidity to the primary procedure^3^ ^-^ ^5^. 

The reported advantages over colostomies and the fact that ileostomy closure complications (20%) and mortality (0.4%) are acceptable have made a temporary diversion a routine after tenuous anastomosis, infected or immunocompromised patients^6^.

The best operative choice for (FAP) patients is complex and usually involve ileorectal anastomosis (IRA) or restorative proctocolectomy with ileal J-pouch anal anastomosis (IPAA). Besides the associated morbidity, IPAA has been considered the procedure of choice as it removes all the colorectal mucosa at risk and leads to good functional results. 

Although not obligatory, IPAA is commonly followed by a loop ileostomy. During a secondary operation, ileostomy takedown is performed as part of a staged procedure that involves potential risks in almost one fifth of patients^7^ ^,^ ^8^. Although some may consider the idea of ileostomy omission in selected patients^9^, we have expressed our fear that the occurrence of pelvic sepsis could act as a trigger to develop desmoid disease after a necessary reoperation^3^ ^,^ ^10^ ^,^ ^11^.

Advantages of the laparoscopic approach in IPAA have been widely described, with special benefits for young patients and for those who may require a further procedure for any reason, as the laparoscopic approach is associated with the formation of less adherences^12^ ^,^ ^13^. 

For this reason, we decided to present our experience with FAP patients treated in a single institution over the last decades. Thus, the present manuscript aimed to analyze the influence of previous surgical approach (laparoscopic or open) on ileostomy takedown related morbidity.

## METHODS

The study was approved by the Gastroenterology Department Ethics Committee (Nº SGP-CPPesq 5812). We retrospectively collected data from FAP patients treated by prophylactic IPAA from 1996 to 2021 in Hospital das Clínicas (University of São Paulo Medical School, Brazil). All patients were operated by staff colorectal surgeons certified by the Brazilian Society of Colorectal Surgeons.

All patients received a two-stage procedure (proctocolectomy, IPAA and diversion). Surgical approach was determined by surgeons’ choice according to the period the patient was treated, individual experience and patient’s clinical features.

Demographic and surgical data were retrieved from patient charts. Patient features such as age, sex, race, previous abdominal surgery before IPAA, surgical approach for IPAA (laparoscopic or open), ileostomy closure data, and types of treatment were analyzed. 

Ileostomy closure was usually performed as a secondary procedure. It was initially attempted through the stoma incision without laparotomy. After a parastomal incision, the loop ileostomy was then dissected and mobilized from surrounding tissues in the abdominal wall till it was completely free. Anastomosis technique varied from a hand-sewn anastomosis to a mechanical one after we started to perform laparoscopic resections. 

After a minor resection of the distal edge thickened bowel (at the proximal and distal limbs), a handsewn anastomosis in an end-to-end fashion was performed with continuous or separated suture. At the surgeon’s discretion, a latero-lateral mechanical anastomosis was also performed using a linear 75 mm stapler to accomplish the Barcelona technique^14^ ^,^ ^15^. In short, the proximal and distal limbs are approximated in parallel, and a linear stapler is introduced through two small enterotomies to create a common channel after firing, by creating a side-to-side anastomosis. Finally, another firing using the same stapler excludes the segment containing the two small perforations and the extremity of the specimen. 

Outcomes of ileostomy closure following laparoscopic and open colorectal resections were compared using chi-square for categorical variables and Student’s *t* test for continuous variables.

## RESULTS

From 1996 to 2021, 88 FAP patients underwent restorative proctocolectomy (RPC) with ileal pouch-anal anastomosis (IPAA). Pouch failure (including permanent stoma) was registered in four patients (6.3 %), being two after laparoscopy (LAP, 5.2%) and two after conventional (OPEN, 8.0%) procedures. Overall, 84 (53 female and 31 male) patients submitted to ileostomy closure at a median age of 30.6 years (11-64) were eligible for the study. Patient’s demographics are presented in Table 1. 

**TABLE 1:** Demographics of 84 FAP patients undergoing ileostomy closure after conventional (OPEN) or laparoscopic (LAP) restorative proctocolectomy.

Variable	21 OPEN	63 LAP	84 patients	***P* value**
	(25%)	(75%)	Total	
Mean age	31.2 (11.9)	28.2 (12.1)	30.6	*P*=0.134^1^
Range (years)	15-64	14-56	11 a 64	
Male	08 M (38.1%)	40 M (63.5%)	31 (36.9%)	(Fisher)
Female	13 F (61.9%)	23 F (36.5%)	53 (63.1%)	
Sex ratio (M:F)	0.61	1.74	0.58	*P*=0.07^2^
Caucasian	18 (85.7%)	49 (77.7%)	67 (79.8%)	(Fisher)
Non-caucasian	3 (14.3%)	14 (22.3%)	17 (20.2%	*P*=0.542^2^
Previous surgery	3 (14.3%)	5 (7.9%)	8 (9.5%)	(Fisher)
				*P*=0.407^2^

FAP: familial adenomatous polyposis.^1^Student’s *t*-test; ^2^Fisher exact test; ^3^chi-square test.

The laparotomy group (OPEN) was formed by 21 patients (25%), all of them undergoing ileostomy closure with handsewn anastomosis. Otherwise, other 63 patients (75%) were operated with laparoscopic techniques (LAP group) and a mechanical anastomosis was performed as described before. Median intervals to perform ileostomy reversal were different between groups (OPEN 98.3 days vs LAP 88.4 days). Concerning demographics, statistical analysis demonstrated no differences among both groups.

Early (n=27; 32.1%) and late (n=8; 9.5%) complications occurred after IPAA. Regarding the approach, there were registered similar rates of complications after proctocolectomy (Table 2). There was no 30-day mortality in the present series. 

**TABLE 2:** Complications after Open and Lap restorative proctocolectomy and after ileostomy closure.

Variable	Open RPC	Lap RPC	Total	***P* value**
	N=21 (25.0%)	N=63 (75%)	N=84 (100%)	
Early complications	7 (33.3%)	20 (31.7%)	27 (32.1%)	*P*=1.000
Late complications	3 (14.2%)	5 (7.9%)	8 (9.5%)	*P*=0.40
Overall complications	10 (47.6%)	25 (39.6%)	35 (41.6%)	*P*=0.61
IC Complications	3 (14.2%)	3 (4.7%)	6/84=7.1%	*P*=0.16
IC Reoperations	2 (9.5%)	2 (3.1%)	4/84=4.7%	*P*=0.259

IC: ileostomy closure.

After ileostomy closure, six patients developed postoperative complications, leading to an overall 7,1% morbidity rate. However, LAP patients developed less complications (4.7% versus 14.2%) when compared to the OPEN one, with no statistical difference (*P*=0.16). 

Morbidity in the OPEN group were represented by small bowel obstruction (2) and fistula due to dehiscence (1). Among LAP patients, there were two fistula cases and one intestinal obstruction. Reoperations to deal with those complications were necessary in two patients of each group, representing 9.5% and 3.1% of both OPEN and LAP groups (*P*=0.259), respectively. These numbers are presented in Tables 2 and 3.

**TABLE 3:** Type of postoperative complications related to ileostomy takedown after OPEN or LAP restorative proctocolectomy.

PO complications	Open RPC N=21	Lap RPC N=63	P
Small bowel obstruction	2 (9.5%)	1 (1.6%)	*P*=0.1528
Dehiscence at stoma closure site	1 (4.7%)	2 (3.2%)	*P*=0.98
Total complications	3 (14.2%)	3 (4.7%)	*P*=0.16
Surgical x medical management	2 (9.5%)	2 (3.1%)	*P*=0.25

RPC: restorative proctocolectomy.

## DISCUSSION

A diverting loop ileostomy is usually confectioned after low anterior resection, coloanal anastomosis, emergent surgery for diverticulitis and restorative proctocolectomy (RPC) with IPAA. After IPAA, ileostomy aims to lessen the impact of anastomotic leak and to avert pelvic sepsis. 

There exists reasonably sound data to support this idea, once intestinal deviation helps to preserve pouch function and survival^7^ ^,^ ^16^. Besides that, a diverting stoma may cause complications such as clinical dehydration, renal failure, wound infection, skin excoriation, parastomal hernia, prolapse and retraction^17^ ^,^ ^18^.

And after having survived a major surgical procedure, these patients face a second challenge to restore intestinal continuity. Traditionally, the reversal through the stomal site is planned to occur 8-12 weeks later, if no postoperative complications (such as of systemic disturbances, fistula or stenosis) have occurred^19^. In some instances, even a delayed period for ileostomy reversal is considered safe^20^. And it has been generally reported higher morbidity rates after ileostomy closure associated with IPAA when compared to low anterior resection^21^. 

In the present manuscript, the authors present their results after ileostomy closure following open or laparoscopic RPC. Ileostomy reversal complications are associated with many factors. Some discussion exists in relation to the appropriate interval between index surgery (RPC) and ileostomy takedown. In this sense, high rates of complications after early closure (7-12 days) have been reported, probably due to incomplete recovery after the initial procedure^5^ ^,^ ^22^. An interval longer than 8.5 weeks was reported to de­crease complications risk^23^. All our patients had their ileostomy takedown performed in a period greater than 12 weeks after the index surgery.

Others have incriminated chronic kidney disease and complications after primary surgery as risk factors^24^. We had only two ileostomy closure complications occurring in association with early morbidity after index surgery. 

Among the so-called alterable risk factors, the relevance of surgical technique is widely recognized. Intraoperative care plays a critical role, so a meticulous and careful dissection of bowel limbs may avoid bowel and mesenteric injury due to local adhesions. Also, nutrition and caliber of the efferent loop must be checked to prevent future stenosis^25^. 

In this multifactorial scenario, discussion about anastomotic techniques turns to be essential. Stoma reversal may be accomplished either with a handsewn sutured end-to-end anastomosis or latero-lateral stapling techniques. So far, trials and reviews comparing these two configurations have failed to reach a consensus regarding the ideal method^26^ ^,^ ^27^. 

While complication rates (including anastomotic leakage) are generally equivalent, the stapled technique is usually considered faster and capable of providing a larger anastomotic lumen^25^ ^,^ ^28^. However, outcomes focusing postoperative bowel obstruction are controversial, as we may find similar rates^29^ or smaller incidence after stapled in comparison to handsewn suture^30^. In this sense, we have preferred to use the Barcelona staple technique in most cases, because it is considered a safe, fast and effective alternative for ileostomy reversal^15^. This stapled side-to-side intestinal anastomosis was originally described by Ravitch, and it has been commonly used to reconstruct intestinal tract after right colectomy or ileostomy reversal^14^ ^,^ ^15^. 

Although ileostomy reversal is usually considered an easy procedure (a “resident kind of operation”), the associated medical and surgical morbidity (11-37%), mortality (0.4%) and reoperation rates (reaching 8%) suggest that this scenario must be discussed with all patients^17^ ^,^ ^28^ ^,^ ^31^ ^,^ ^32^. Especially after RPC, almost one third of patients may experience complications^28^ ^,^ ^33^ ^,^ ^34^. These considerable outcomes raise the question if they could eventually reduce the benefits of temporary fecal diversion after RPC^32^. 

In the present series, results after ileostomy reversal (7.1% complications and almost 5% reoperations) are in complete accordance with the literature^32^. And when we compared outcomes from the two approaches, we found better numerical results (14.2% x 4.7% complication and 9.5% x 3.1% reoperations rates) among those who have undergone a laparoscopic resection at the first procedure. These results didn’t reach statistical significance probably because of the small number of events in each group.

Besides OPEN and LAP patients had been respectively treated with handsewn and mechanical anastomosis, we don’t credit these different results to this fact, cause comparative studies and meta-analysis showed similar complication rates with both techniques^30^ ^,^ ^35^ ^,^ ^36^ ^,^ ^37^. Conversely, this difference may be attributable to the greater incidence of small bowel obstruction after conventional resection (9.5% x 1.6%). As an eventual consequence, the need of surgery to treat complications among these patients was also greater (9.5% x 3.1%).

The time trend and relevance of surgical approach to postoperative outcomes have made laparoscopic surgery the preferred choice for colorectal resections. Over a 13-years period in Japan, Ueno et al.^38^ reported that laparoscopic surgery reached 74% of the preferences in FAP patients. Since we started to perform laparoscopic total colectomies in 2003 in our hospital, only four patients were operated via laparotomy, mainly due to previous open surgery or obesity. This preference and experience have resulted in many publications concerning this issue.

The influence of surgical approach on adhesions formation after RPC has been a matter of controversial debate in the literature^39^ ^,^ ^40^ ^,^ ^41^, but besides not being supported by high-grade evidence, the laparoscopy potential to cause less adhesions leading to reoperations is well accepted^42^ ^,^ ^43^. In ulcerative colitis patients, those undergoing laparoscopic RPC were found to develop fewer incisional, abdominal and adnexal adhesions when compared to those with previous laparotomy^40^.

So far, the relation between surgical approach with ileostomy takedown complications have deserved little interest in the literature^44^ ^,^ ^45^. In a pioneer study comparing stoma-related complications after laparoscopic and open IPAA for UC, there were not found significant differences in this regard^44^. In another report, however, patients previously operated via laparoscopic approach developed less postoperative complications. In this study, RPC and IPAA represented 60% of all 351 patients^45^.

The present single-center study is certainly limited by its retrospective nature. But it represents another report of our institution to analyse the potential merits and advantages of using minimally invasive techniques during major colorectal resections^12^. In our series, loop ileostomy reversal in those previously operated through laparoscopic procedures were associated with numerical reduced morbidity, need for reoperation and with a trend to manage complications medically. Probably, the appreciation of a greater number of cases in the future will turn possible to reveal a statistical difference between these two groups of patients.

In a complex patient such as those with FAP, it is essential to assume operative decisions aiming to lessen surgical complications and the risk of abdominal sepsis and reoperations after restorative proctocolectomy. The reason is that surgical trauma may be the trigger to develop desmoid disease, an important cause of death within this population^10^ ^,^ ^11^. Thus, in a context of high-quality medical management, we think that the laparoscopic approach should be the preferable option for skilled surgeons dealing with this young population that are frequent candidates to complex surgical procedures and reoperations throughout life. 

## CONCLUSION

In the condition of the present manuscript, the abdominal approach used for restorative proctocolectomy doesn’t seem to decisively influence outcomes after loop ileostomy reversal. The numerical differences we found between OPEN and LAP groups suggests that a future analysis of a greater number of patients may probably demonstrate an expected greater complication and reoperation rates in those previously treated through OPEN procedures.
